# Study on Viscoelastic Properties of Asphalt Mixtures Incorporating SBS Polymer and Basalt Fiber under Freeze–Thaw Cycles

**DOI:** 10.3390/polym12081804

**Published:** 2020-08-11

**Authors:** Wensheng Wang, Guojin Tan, Chunyu Liang, Yong Wang, Yongchun Cheng

**Affiliations:** 1 College of Transportation, Jilin University, Changchun 130025, China; wangws17@mails.jlu.edu.cn (W.W.); tgj@jlu.edu.cn (G.T.); chengyc@jlu.edu.cn (Y.C.); 2 Jilin Province Highway Administration Bureau, Changchun 130021, China; wangyjlgl@gmail.com

**Keywords:** asphalt mixture, basalt fiber, viscoelastic characteristics, freeze–thaw damage

## Abstract

This study aims to study the viscoelastic properties of asphalt mixtures incorporating styrene–butadiene–styrene (SBS) polymer and basalt fiber under freeze–thaw (F-T) cycles by using the static creep test. Asphalt mixture samples incorporating styrene–butadiene–styrene (SBS) polymer and basalt fiber were manufactured following the Superpave gyratory compaction (SGC) method and coring as well as sawing. After 0 to 21 F-T cycles processing, a uniaxial compression static creep test for the asphalt mixture specimens was performed to evaluate the influence of F-T cycles. The results indicated that the F-T cycles caused a larger creep deformation in the asphalt mixtures, which led to a decrease in the rut resistance of the asphalt mixtures incorporating SBS polymer and basalt fiber. Besides, the resistance to deformation decreased significantly in the early stage of F-T cycles. On the other hand, the viscoelastic parameters were analyzed to discuss the variation of viscoelastic characteristics. The relaxation time increased with F-T cycles, which will not be conducive to internal stress dissipation. Compared with lignin fiber, basalt fiber can improve the resistance to high-temperature deformation and the low-temperature crack resistance of asphalt mixtures under F-T cycles.

## 1. Introduction

Asphalt pavement as an important part of transportation infrastructure plays a significant role in social development. With the increase of traffic demand, ordinary asphalt pavements often fail to meet the performance requirements, resulting in a few destructions including low and medium-temperature cracking, high-temperature rutting, freeze–thaw (F-T) destruction, and so on [1,2,3,4]. To improve mechanical performances, various additives such as rubbers, polymers, fibers, and other additive materials have been adopted to incorporate with asphalt [5,6,7,8]. Studies demonstrated that polymers have been proved to improve the high-temperature rutting, moisture damage, etc. [9]. Besides, adding fibers to asphalt mixtures usually increases the mechanical performances such as cracking resistance [10]. To improve the compressive capabilities of asphalt materials effectively, researchers have made lots of efforts and tried many novel additives.

Styrene–butadiene–styrene (SBS) polymer has been proved to improve asphalt well due to its dual characteristics of rubber and plastic [11,12,13]. Today, the SBS polymer modifiers are commonly applied in modified bitumen, and the demand for SBS modifiers is increasing with the development of highway construction. Imaninasab [14] investigated and evaluated the influences of the modification process of two kinds of polymers on anti-rutting performance at a high temperature of stone mastic asphalt (SMA). Wang et al. [15] explored the experimental methods of polymer-modified asphalt systematically so as to ensure the quality and requirement of construction engineering in asphalt pavement. Hajikarimi et al. [16] investigated the rheological and mechanical performances of SBS-modified asphalt as well as its binder with three proportions by using rheometer equipment for the purpose of analyzing the storage and loss modulus and viscoelastic behavior. Furthermore, they used the finite element software to simulate the viscoelastic behavior of neat and SBS-modified asphalt, respectively [17].

Basalt fiber as a novel environmentally friendly mineral fiber has several excellent advantages including better strength, high temperature, as well as acid and alkali resistance [18]. In recent years, basalt fiber has gained more and more attention for improving asphalt mixtures [19,20]. Studies demonstrated that basalt fibers have a greater impact on enhancing the comprehensive performance of asphalt materials to some extent. Sun et al. [21] explored an enhancement impact of basalt fiber on the toughness of asphalt materials. Qin et al. [22] investigated the influences of basalt fibers with various sizes and contents on asphalt mastics and compared them with other common fibers. Li et al. [23] conducted three-point bending tests at three low temperatures on asphalt mixtures with various basalt fiber contents and proposed a distinction method of fracture type based on the bending coefficient. In view of the good improvement effects of SBS and basalt fiber on different aspects of asphalt, most researchers tried to employ the incorporation of polymers and fibers into asphalt mixtures to improve its comprehensive performance. Gu et al. [24] pointed out that SBS-modified asphalt reinforced with basalt fiber has a higher rutting factor compared with original asphalt based on dynamic shear rheological tests and the repeated creep tests. Tanzadeh et al. [25] investigated the open-graded friction course modified by polymer and basalt fiber by the drainage test and common mechanical test. Miao et al. [26] examined four types of fibers (i.e., fiber-reinforced plastic, two lignin fibers, and basalt fiber) and four types of asphalt including neat asphalt and polymers-modified asphalt based on the interfacial properties. Luo et al. [27] evaluated the enhancement impact of SBS and basalt fiber on the anti-rutting and anti-cracking of asphalt mixtures by the Hamburg wheel track test and low-temperature bending test, respectively. Kou et al. [28] used basalt fiber to reinforce SBS-modified asphalt, and they found that SBS-modified asphalt with basalt fiber can make use of both advantages of additives.

To better apply asphalt mixtures to the northeast seasonal frozen regions, a series of studies have been carried out toward exploring the F-T destruction characteristics of asphalt mixtures [29,30,31,32]. In addition, many scholars conducted various experiments of asphalt mixtures under varying F-T actions to explore the damage evolution [33,34,35]. Tarefder et al. [36] analyzed the influences of F-T action on the fatigue and rheometer of asphalt mixtures using several experimental methods. For asphalt mixtures with basalt fiber incorporating SBS and fiber, Liang et al. [37] investigated its fracture characteristics and analyzed the mechanical performance under the action of F-T based on the acoustic emission method. Fan et al. [38] quantitatively evaluated the fatigue performance of asphalt mixtures under the repeated F-T action. Cheng et al. [39] made an overall assessment of the mechanical properties of asphalt mixtures with basalt fiber and analyzed the improvement impact of F-T resistance based on volumetric and mechanical parameters [40]. Cheng et al. [41] established a damage evolution of the mechanical performance of asphalt mixtures exposed to repeated F-T actions through reliability and damage theory and predicted and analyzed its internal damage degradation. Badeli et al. [42] explored the influences of F-T actions on the fatigue cracking of asphalt mixtures considering seasonal ambient temperature variations. Eric et al. [43] evaluated the moisture stability and performance degradation of asphalt mixtures subjected to F-T action and studied the viscoelastic behavior of asphalt mixtures.

According to the above studies, it is known that there are many studies on the performance of SBS polymer-modified asphalt mixtures incorporating basalt fiber and the evaluation of F-T damage of asphalt mixtures. However, at present, most studies focused on the conventional pavement performances of asphalt mixtures but often ignored its viscoelastic mechanics. Hence, this study aims to analyze the viscoelastic mechanical characteristics (creep and relaxation) for asphalt mixtures incorporating SBS polymer and basalt fiber. The viscoelastic models based on a uniaxial compression creep test were established. Then, the corresponding viscoelastic model parameters were analyzed to discuss the influences of F-T cycles and test temperatures on the variation of viscoelastic characteristics for asphalt mixtures.

## 2. Experimental Materials and Methods

### 2.1. Raw Materials and Specimen Preparation

#### 2.1.1. Experimental Materials

**Figure 1:**
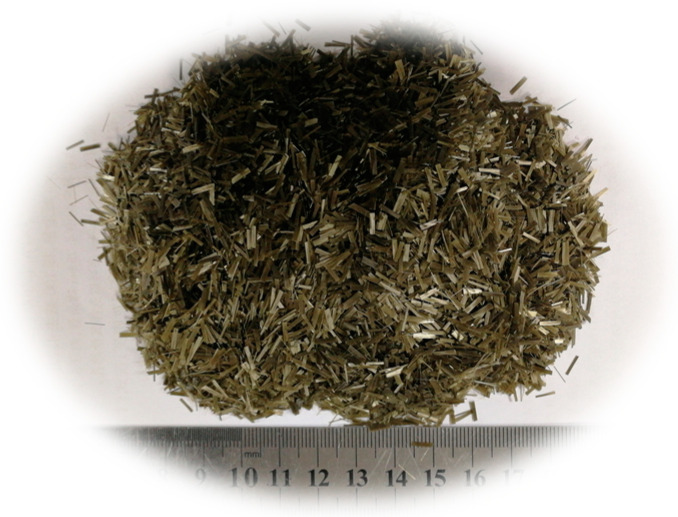
Basalt fiber with length of 6 mm used in this study.

SBS-modified asphalt, as one of the most commonly used asphalt types, was selected in this study, which was from Yingkou, China. Crushed basalts were used as the aggregate type and limestone powder was used as the filler, which were provided by Jiutai City and Siping City, China, respectively. Then, the basalt fiber shown in Figure 1 was employed as a fiber stabilizer. The technical properties of all the above materials refer to Table 1, Table 2, Table 3, Table 4 and Table 5 [35,37].

#### 2.1.2. Specimen Preparation

The asphalt mixture type stone mastic asphalt (SMA) was employed in this study. As known, SMA is a widely used asphalt mixture type composed of high-content coarse aggregate, mineral powder, and asphalt as well as low-content fine aggregate, which was initially formed in Germany in the 1960s and first applied in China in 1992 [8]. Due to the better resistance to deformation and durability, SMA has been extensively applied for most pavement surfaces of highways in China. The median gradation of SMA-13 was employed as the gradation curve for manufacturing samples [8].

**Figure 2:**
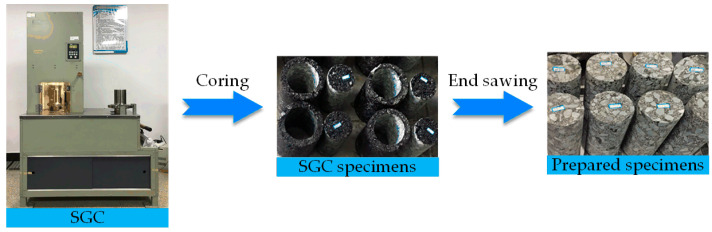
Specimen preparation procedure in this study.

To better simulate the actual pavement construction, the Superpave gyratory compaction (SGC) method was selected to prepare asphalt mixtures incorporating SBS polymer and basalt fiber in this study, which is a novel Superpave mixture design and molding method. It has been proved that the internal structures of SGC asphalt mixture samples are consistent with core specimens from an actual road with a good correlation, which have less porosity variability. The optimal asphalt–aggregate ratio was determined as 5.7% and basalt fiber content was chosen as 0.34% by the mass of SBS-modified asphalt [8]. The detailed procedure of specimen preparation has been described, and the set SGC parameters can be found in the previous studies [8,41]. The sample preparation procedure followed the Chinese standard JTG E20-2011 [44]. As shown in Figure 2, asphalt mixture specimens were first manufactured following the SGC method. After de-molding and cooling, asphalt mixture samples (diameter: 100 mm, height: 150 mm) described in the standard JTG E20-2011 T0738 could be prepared through using a core drilling machine and a cutting machine [5].

### 2.2. Experimental Procedure and Test Configuration

#### 2.2.1. Experimental Process

**Figure 3:**
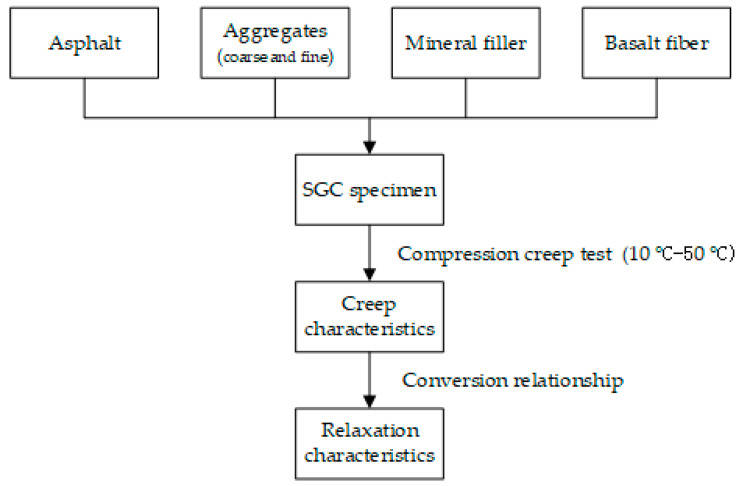
Experimental process flowchart.

Samples of asphalt mixtures incorporating SBS polymer and basalt fiber were firstly manufactured and molded following the SGC method in this study. After that, before the performance test, samples were treated by 0 to 21 F-T cycles, respectively. The detailed procedure of F-T cycle processing for specimens has been described in the previous study [41]. Then, the static creep test was conducted under uniaxial compression loadings at five temperatures; the detailed experimental process was reported in the previous study [45]. The experimental process flowchart is presented in Figure 3.

#### 2.2.2. Protocol of Uniaxial Compression Creep Test

**Figure 4:**
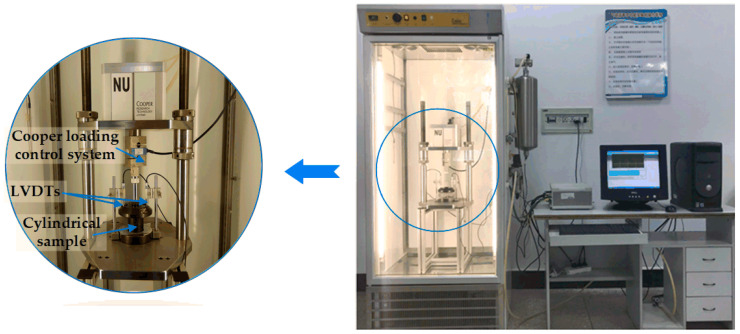
Uniaxial static compression creep test by Cooper NU-14 in this study.

The creep test is the most common test method for the viscoelastic mechanical behavior of asphalt mixtures. The uniaxial compression creep test is the easiest to achieve and most widely used viscoelastic test; thus, it was employed to study the viscoelastic mechanical behavior of asphalt mixtures in the experimental design. In this experiment, a servo-pneumatic universal testing machine (NU-14, Cooper Technologies Ltd., Ripley, UK) with a stroke of 30 mm was employed to perform a uniaxial compression creep test [45]. The NU-14 with an environmental chamber has a frequency of up to 30 Hz, and it is controlled within −10 to 60 °C. Before the uniaxial compression creep test, equipped specimens were kept for at least 5 h in a testing chamber to equilibrate to the specified condition, as illustrated in Figure 4. Two linear variable differential transformer (LVDT) brackets were placed to asphalt mixture specimens, as shown in Figure 4.

For asphalt mixtures incorporating SBS polymer and basalt fiber under varying F-T actions, the test specimen with poly tetra fluoroethylene films placed at the top and bottom ends was applied to a static compressive force with a stress level of 0.3 MPa for 3600 s at specific conditions (10 °C, 20 °C, 30 °C, 40 °C, 50 °C) during the uniaxial compression creep test. The two vertical LVDTs could record the real-time axial strains of asphalt mixture specimens, which would be used to calculate the creep strain varying with loading time. For each test, two SGC core specimens were used as parallel tests, and the average of the test results was analyzed.

### 2.3. Theory of Viscoelastic Mechanics of Asphalt Mixture

#### 2.3.1. Basic Theory of Viscoelastic Model

An asphalt mixture is a typical viscoelastic material with both elastic and viscous behaviors. When describing the viscoelastic behavior of asphalt mixtures, the spring element is used to represent Hook elasticity, the viscous pot element describes Newtonian viscosity, and the combination of spring element and viscous pot element is utilized to reflect its general viscoelasticity.

Regarding the Hook elasticity represented by the spring element and Newtonian viscosity by the viscous pot element, the stress (*σ*)–strain (*ε*) relationships (in Equations (1) and (2)) follow Hooke’s law and Newton’s internal friction law, respectively [46]:

*σ = E·ε*, (1)

(2) $$\sigma =\eta \cdot \dot{\varepsilon },$$

where *E* is the elastic modulus, and *η* is the viscosity coefficient.

**Figure 5:**
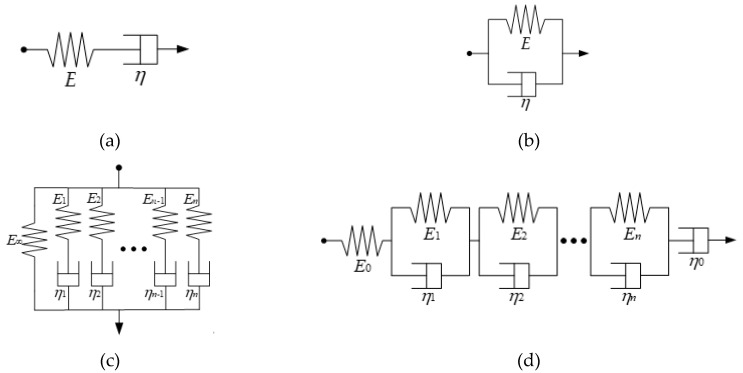
Viscoelastic models: (**a**) Maxwell element; (**b**) Kelvin element; (**c**) generalized Maxwell model; and (**d**) generalized Kelvin model.

The characteristics of the general viscoelasticity represented by the combination of spring element and viscous pot element are between the above two. The two most basic elements in the viscoelastic constitutive model (in Figure 5a,b) can be obtained by connecting springs and viscous pot elements in series and parallel, namely the Maxwell element and Kelvin element. The corresponding stress (*σ*)–strain (*ε*) relationships are shown in Equations (3) and (4), respectively:

(3) $$\dot{\varepsilon }=\dot{\sigma }/E+\sigma /\eta ,$$

(4) $$\sigma =E\cdot \varepsilon +\eta \cdot \dot{\varepsilon },$$

where the Maxwell element reflects the stress relaxation phenomenon of viscoelastic material, the relaxation modulus of the Maxwell element is *E*·exp(−*Et*/*η*), the Kelvin element reflects the creep delay phenomenon of the viscoelastic material, and the creep compliance of the Kelvin element is 1/*E*·(1−exp(−*Et*/*η*)).

#### 2.3.2. Generalized Maxwell Model and Generalized Kelvin Model

A single Maxwell model or Kelvin model can characterize the mechanical properties of viscoelastic materials to a certain extent. However, because the elements are too simple, it is difficult to accurately reflect the complex viscoelastic mechanical behavior of asphalt mixtures. In order to better use the viscoelastic model and theory, several Maxwell models or Kelvin models in parallel or in series are used to form a generalized Maxwell model or a generalized Kelvin model, as shown in Figure 5c,d.

At present, the generalized Maxwell model is widely used to simulate the stress relaxation behavior of viscoelastic materials, and the generalized Kelvin model is used to simulate the creep behavior of viscoelastic materials. Generally, the relaxation modulus (*E*(*t*)) in Equation (5) and creep compliance (*J*(*t*)) in Equation (6) are used to characterize the viscoelastic behavior of viscoelastic materials [45].

*E(t) = σ(t)/ε_0_*, (5)

*J(t) = ε(t)/σ_0_*, (6)

where *σ*_0_ and *ε*_0_ are the constant stress and constant strain, respectively.

In general, the form of Prony series is used to characterize the complex stress relaxation behavior described by the generalized Maxwell model and the creep behavior described by the generalized Kelvin model. Then, the relaxation modulus (*E*(*t*)) of the generalized Maxwell model and creep compliance (*J*(*t*)) of the generalized Kelvin model are expressed as:

(7) $$E(t)=E_{\infty }+\sum_{i=1}^{n}E_{i}e^{-\frac{t}{\rho_{i}}},$$

(8) $$J(t)=J_{0}+\sum_{i=1}^{n}J_{i}(1-e^{-\frac{t}{\tau_{i}}}),$$

where *E*_∞_ is the equilibrium relaxation modulus, *J*_0_ is the instantaneous flexibility modulus, *t* is the creep loading time, *ρ_i_* is the relaxation time, and *τ_i_* is the delay time.

#### 2.3.3. Conversion between Relaxation Modulus and Creep Compliance

The linear superposition principle (Boltzmann superposition principle) is a basic viscoelastic mechanics theory proposed by Boltzmann. For viscoelastic materials, the stress–strain relationship in a linear viscoelastic state can be expressed according to the Boltzmann superposition principle [45]:

(a) Controlled by stress (*σ*(*t*)), the strain response (*ε*(t)) is expressed mathematically:

(9) $$\varepsilon (t)=\int_{-\infty }^{t}\frac{d\sigma (\tau )}{d\tau }J(t-\tau )d\tau ,$$

where *J*(*t*) is the creep compliance of viscoelastic material, and *τ* is a dummy variable.

(b) Controlled by strain (*ε*(*t*)), the stress response (*σ*(*t*)) expressed mathematically:

(10) $$\sigma (t)=\int_{-\infty }^{t}\frac{d\varepsilon (\tau )}{d\tau }E(t-\tau )d\tau ,$$

where *E*(*t*) is the relaxation modulus of viscoelastic material.

According to the viscoelasticity principle, Laplace transform is performed by Equations (9) and (10) respectively, and the viscoelastic relationship in the time domain can be converted in the Laplace domain:

(11) $$\tilde{\varepsilon }(s)=s\tilde{J}(s)\tilde{\sigma }(s),$$

(12) $$\tilde{\sigma }(s)=s\tilde{E}(s)\tilde{\varepsilon }(s),$$

where $\tilde{\sigma }(s)$, $\tilde{\varepsilon }(s)$, $\tilde{J}(s)$, and $\tilde{E}(s)$ are the stress, strain, creep compliance, and relaxation modulus in the Laplace domain, respectively.

From the above Equations (11) and (12), the conversion relationship between creep compliance and relaxation modulus in the Laplace domain can be obtained:

(13) $$\tilde{E}(s)\tilde{J}(s)=\frac{1}{s^{2}}.$$

By using the Laplace inversion, the relationship between creep compliance and relaxation modulus in the time domain can be obtained:

(14) $$\int_{0}^{t}E(t-\tau )J(\tau )d\tau =t,$$

(15) $$\int_{0}^{t}E(\tau )J(t-\tau )d\tau =t.$$

The essence of transforming creep compliance into a relaxation modulus is that the generalized Kelvin model used in the creep test is converted into the generalized Maxwell model in the relaxation test.

## 3. Results and Discussion

### 3.1. Creep Characteristics of Asphalt Mixtures Incorporating SBS Polymer and Basalt Fiber under F-T Cycles

#### 3.1.1. Creep Compliance Curve of Asphalt Mixtures Incorporating SBS Polymer and Basalt Fiber under F-T cycles

**Figure 6:**
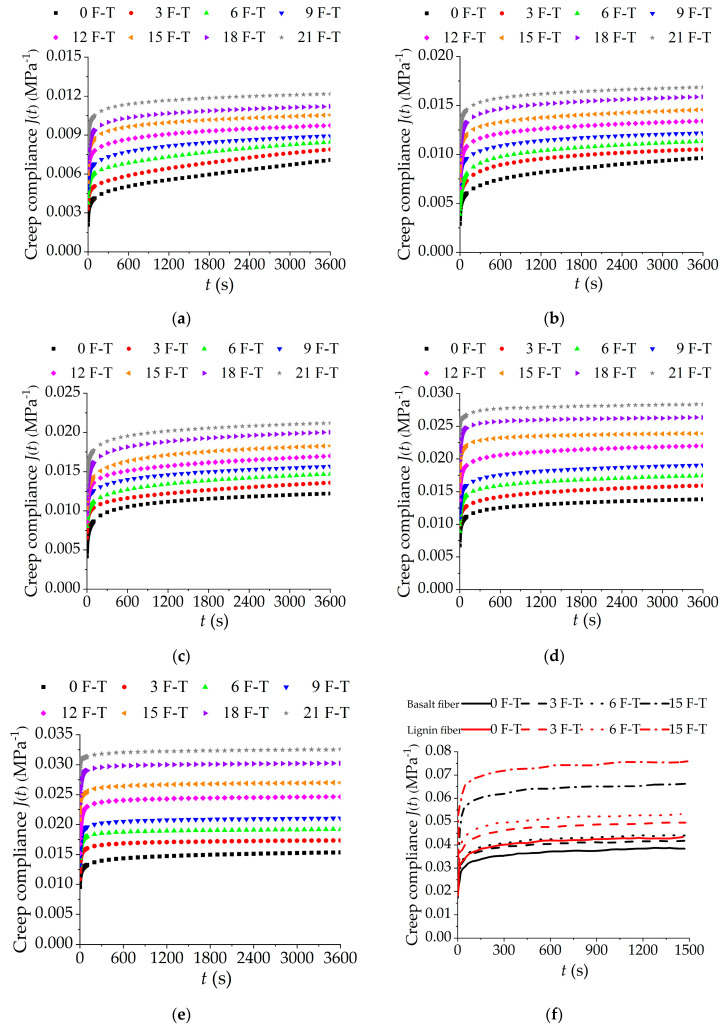
Creep compliances of asphalt mixtures under F-T cycles at various temperatures: (**a**) 10 °C; (**b**) 20 °C; (**c**) 30 °C; (**d**) 40 °C; (**e**) 50 °C; and (**f**) comparison at 50 °C.

The viscoelastic properties of asphalt mixtures incorporating SBS polymer and basalt fiber are of great significance to its performance analysis and engineering applications. The creep test is an effective test method for evaluating the viscoelastic properties of asphalt mixtures. In this study, to analyze in detail the change of creep characteristics of asphalt mixtures incorporating SBS polymer and basalt fiber under F-T cycles, the axial strain and stress of asphalt mixtures incorporating SBS polymer and basalt fiber can be recorded with the help of a servo-pneumatic universal testing machine NU-14 based on the uniaxial compression creep test. According to Equation (6), the creep compliance results of asphalt mixtures incorporating SBS polymer and basalt fiber under F-T cycles at different test temperatures can be calculated. Figure 6a–e present the creep compliance curves versus time under five different test temperatures (10 °C, 20 °C, 30 °C, 40 °C, 50 °C) and F-T cycles (0, 3, 6, 9, 12, 15, 18, 21).

As observed in Figure 6, at the same temperature, the creep compliance of asphalt mixtures incorporating SBS polymer and basalt fiber increases with the increasing number of F-T cycles. The increasing creep compliance at the same loading stress level means that the strain of asphalt mixture specimens increases with the increasing number of F-T cycles. By comparing Figure 6e,f, it can be found that the creep compliance of asphalt mixture specimens under F-T cycles are ordered from small to large as follows: asphalt mixture SGC specimens reinforced with basalt fiber < asphalt mixture Marshall specimens reinforced with basalt fiber < asphalt mixture Marshall specimens reinforced with lignin fiber.

The increasing creep compliance shows that the rutting resistance of asphalt mixtures incorporating SBS polymer and basalt fiber would be reduced by the action of F-T cycles. At the same time, the upward shift of the creep compliance curve increases slightly with the increase of F-T cycles. The reason is that the effect of F-T cycles exacerbates the deterioration of creep properties of asphalt mixtures incorporating SBS polymer and basalt fiber. In addition, to illustrate the reinforcement effect of basalt fiber on asphalt mixtures, the uniaxial compression creep results at 50 °C of asphalt mixture Marshall specimens with lignin fiber and basalt fiber in the previous study [47] were chosen as the control group, which is plotted in Figure 6f. The comparison result means that the addition of basalt fiber is beneficial to improve the creep performance of the asphalt mixtures, and it is more obvious in its ability to resist F-T damage.

#### 3.1.2. Influence Analysis of F-T Cycles on Creep Characteristics of Asphalt Mixtures Incorporating SBS Polymer and Basalt Fiber

1. Analysis of Creep Behavior of Asphalt Mixtures Incorporating SBS Polymer and Basalt Fiber Based on Generalized Kelvin Model

The generalized Kelvin model is composed of Kelvin models connected in parallel, which has been widely used to simulate the creep behavior of viscoelastic materials, and it can well describe the creep behavior of asphalt mixtures. The Prony series is a commonly used method to characterize the viscoelastic properties of asphalt mixtures and the Prony series of creep compliance of the asphalt mixtures is formulated in Equation (8). In this section, the Prony series is used to fit the creep compliance curve of asphalt mixture specimens incorporating SBS polymer and basalt fiber under various F-T cycles. According to the fitting model, the fitting parameters of the Prony series of the asphalt mixtures incorporating SBS polymer and basalt fiber at 10 °C, 20 °C, 30 °C, 40 °C, and 50 °C can be obtained, which are listed in Table 6, Table 7, Table 8, Table 9 and Table 10.

From Table 6, Table 7, Table 8, Table 9 and Table 10, it can be seen that the fitting coefficient *R*^2^ values of the Prony series of asphalt mixtures incorporating SBS polymer and basalt fiber at each test temperature are greater than 0.99 and close to 1. At the same time, by comparing the parameters of the generalized Kelvin model under different F-T cycles at the same test temperature, it can be seen that the fitting parameters of the generalized Kelvin model show an increasing trend as a whole as the number of F-T cycles increases.

The generalized Kelvin model has a good fitting effect and can be used to accurately describe the creep behavior and development trend of asphalt mixtures. However, due to too many parameters of the generalized Kelvin model, it is difficult for the fitting model parameters to accurately reflect the variation of the viscoelastic properties of asphalt mixtures incorporating SBS polymer and basalt fiber under the action of F-T cycles.

2. Analysis of Creep Behavior of Asphalt Mixtures Incorporating SBS Polymer and Basalt Fiber Based on Burgers Model

The Burgers model is a more commonly used viscoelastic model for asphalt mixtures, which not only ensures accuracy but it is also relatively simple and practical [8]. The Burgers model parameters include the elastic modulus (*E*_1_) and the viscosity coefficient (*η*_1_) of the Maxwell model, as well as the elastic modulus (*E*_2_) and the viscosity coefficient (*η*_2_) of the Kelvin model. The four parameters of the Burgers model for the creep compliance of the asphalt mixtures can better reflect the variation of the viscoelastic properties of the asphalt mixtures varying with test temperature, which is difficult to achieve in the generalized Kelvin model. In order to further analyze the effect of F-T cycles on the viscoelastic properties of asphalt mixtures incorporating SBS polymer and basalt fiber, the Burgers model was used to fit the uniaxial compression creep compliance of asphalt mixture SGC specimens at various test temperatures under different F-T cycles. The Levenberg–Marquardt (LM) method and general global optimization method were used as the fitting methods. According to the fitting model, the four parameters of the Burgers model of asphalt mixture specimens incorporating SBS polymer and basalt fiber at five temperatures (10 °C, 20 °C, 30 °C, 40 °C, and 50 °C) can be obtained, and the delay time of test specimens can be also calculated, which are shown in Table 11, Table 12, Table 13, Table 14 and Table 15.

From Table 11, Table 12, Table 13, Table 14 and Table 15, it can be seen that the fitting coefficient *R*^2^ values of the Burgers model of asphalt mixtures incorporating SBS polymer and basalt fiber at each test temperature are greater than 0.9. The fitting coefficient *R*^2^ values of the Burgers model are slightly smaller than those of the generalized Kelvin model. However, the Burgers model is still in good agreement with the creep compliance, which can be used to describe the variation trend of creep compliance of asphalt mixtures varying with F-T cycles and test temperatures.

**Figure 7:**
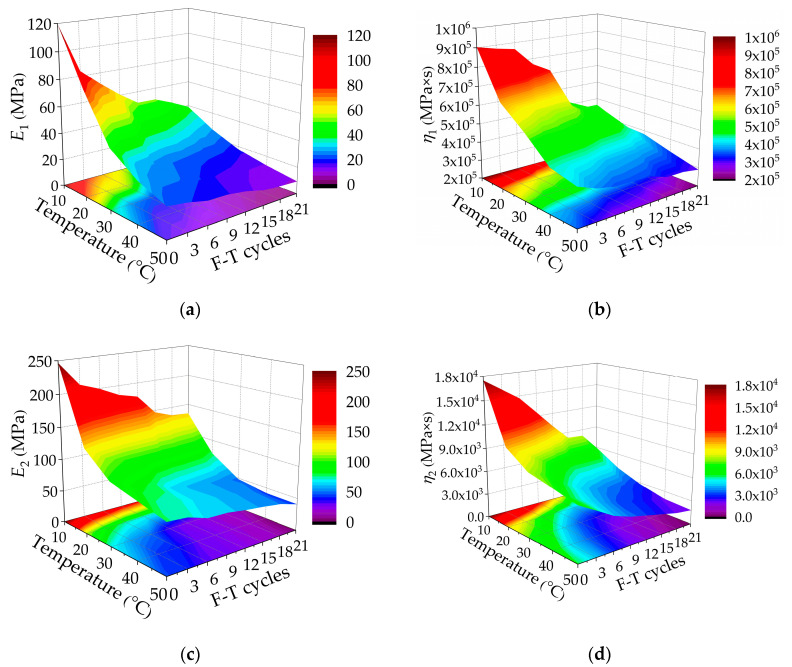

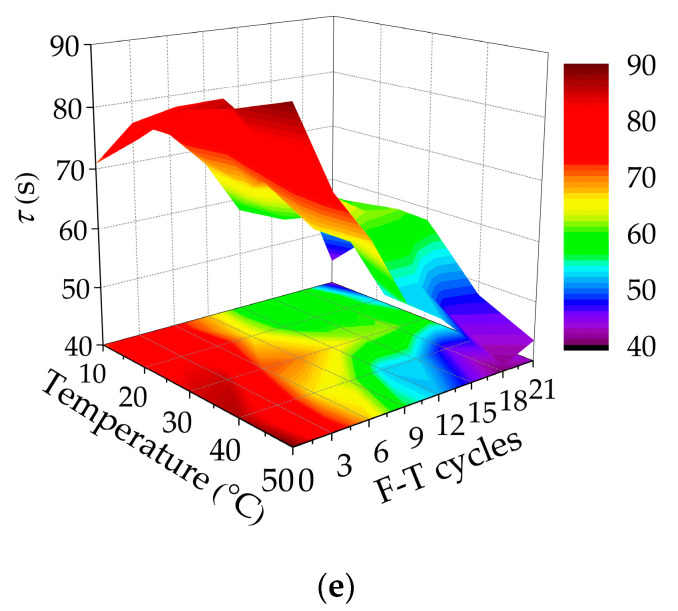
Burgers model parameters of creep compliances varying with temperatures and F-T cycles: (**a**) *E*_1_; (**b**) *η*_1_; (**c**) *E*_2_; (**d**) *η*_2_; and (**e**) *τ*.

The Burgers model parameters are used to further analyze the viscoelastic properties of the asphalt mixtures. The variations of the four creep parameters and delay time of the Burgers model of the asphalt mixtures incorporating SBS polymer and basalt fiber with test temperature and F-T cycles are shown in Figure 7. It can be seen that the instantaneous elastic modulus *E*_1_, instantaneous viscosity coefficient *η*_1_, delayed elastic modulus *E*_2_, and delayed viscosity coefficient *η*_2_ of the Burgers model of asphalt mixtures incorporating SBS polymer and basalt fiber decrease varying with the test temperature increasing, while the delay time τ increases slightly. Similarly, at the same test temperature, it can be seen that the instantaneous elastic modulus *E*_1_, instantaneous viscosity coefficient *η*_1_, delayed elastic modulus *E*_2_, and delayed viscosity coefficient *η*_2_ of the Burgers model show a downward trend as the F-T cycle increases, and the delay time *τ* increases slightly.

Although the change rule of the Burgers model parameters of the asphalt mixtures under F-T cycles is similar to that variation with test temperature, the reasons are different. The sensitivity of the asphalt mixtures to temperature also decreases with F-T cycles. The Burgers model parameters decreased significantly in different degrees from 0 to 12 F-T cycles; however, after 12 or 15 F-T cycles, the variation of the Burgers model parameters gradually slows down. This is because in the early stage of the F-T cycles, the skeleton structure of the asphalt mixtures is affected by the sudden frost force, which leads to an obvious pore change, increasing the porosity and connecting more pores. Thus, the skeleton structure of the asphalt mixtures changes significantly. The decay rate of creep performance slows down with F-T cycles. In the late stage of F-T cycles, the cohesion between aggregates in the asphalt mixtures is weakened due to the intrusion of water.

### 3.2. Relaxation Characteristics of Asphalt Mixtures Incorporating SBS Polymer and Basalt Fiber under F-T Cycles

#### 3.2.1. Relaxation Modulus Curve of Asphalt Mixtures Incorporating SBS Polymer and Basalt Fiber under F-T Cycles

**Figure 8:**
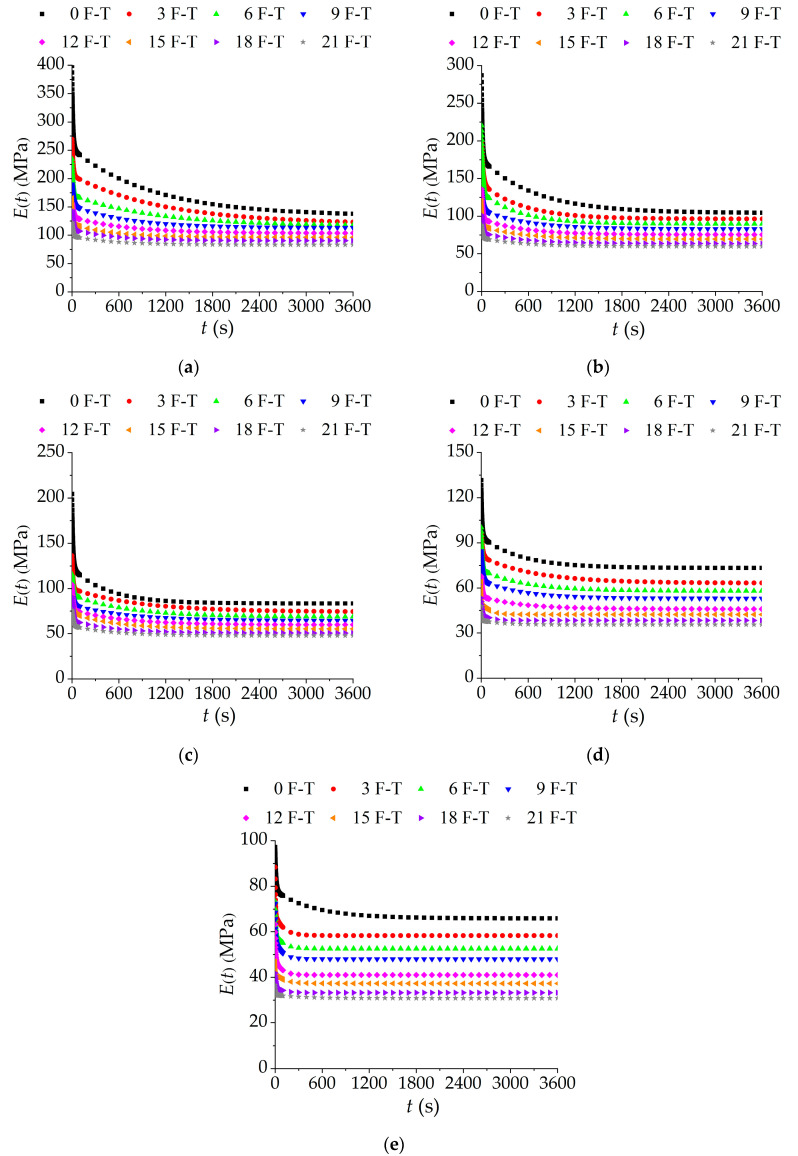
Relaxation moduli of asphalt mixtures under F-T cycles at various temperatures: (**a**) 10 °C; (**b**) 20 °C; (**c**) 30 °C; (**d**) 40 °C; and (**e**) 50 °C.

In view of the fact that creep and relaxation belong to the two aspects of the viscoelastic properties of asphalt mixtures, to comprehensively analyze the variation of viscoelastic properties of asphalt mixtures incorporating SBS polymer and basalt fiber under F-T cycles, it is necessary to analyze the relaxation properties. According to the conversion relationship in Equations (13)–(15), the relaxation modulus can be obtained from the conversion of creep compliance for asphalt mixtures incorporating SBS polymer and basalt fiber under F-T cycles at various temperatures (10 °C, 20 °C, 30 °C, 40 °C, 50 °C), as shown in Figure 8.

It can be seen from Figure 8 that the relaxation modulus of asphalt mixtures under F-T cycles decreases with the extension of loading time. This is because the internal stress of the asphalt mixtures is gradually dissipated; i.e., it is a stress relaxation phenomenon. At the same test temperature, the relaxation modulus curve of the asphalt mixtures incorporating SBS polymer and basalt fiber gradually moves down with the decreasing number of F-T cycles. This is attributed to the increasing weakness between the aggregates and asphalt under F-T cycles. It is beneficial to the low-temperature crack resistance from the perspective of relaxation. However, it is unreliable only considering the relaxation modulus curve, and it is often necessary to comprehensively consider several relaxation parameters for analysis.

By transforming the relaxation modulus of the asphalt mixtures with double logarithmic coordinates and then fitting with a linear equation, the change rate of the relaxation modulus can be obtained and is shown in Table 16, which means that there is a good linear fitting effect. From this perspective, it can reflect the relaxation rate and relaxation time of the asphalt mixtures and it can also be used to intuitively evaluate the relaxation performance of the asphalt mixtures incorporating SBS polymer and basalt fiber. According to Table 16, the slope of the relaxation modulus of the asphalt mixtures decreases, and the relaxation time is also prolonged, which is not conducive to the dissipation of internal stress; then, the relaxation ability of the asphalt mixtures is reduced. At the same time, the relaxation performance of the asphalt mixture Marshall specimens with lignin fiber in the previous test was compared with the relaxation results in this study. It can be seen that the addition of basalt fiber is conducive to its relaxation performance and enhances the resistance to cracks.

#### 3.2.2. Influence Analysis of F-T Cycles on Relaxation Characteristics of Asphalt Mixtures Incorporating SBS Polymer and Basalt Fiber

1. Analysis of Relaxation Behavior of Asphalt Mixtures Incorporating SBS Polymer and Basalt Fiber Based on Generalized Maxwell Model

The generalized Maxwell model can fit the relaxation modulus of the asphalt mixtures well and accurately describe the change trend of relaxation behavior. Then, the generalized Maxwell model is used to analyze the relaxation modulus of the asphalt mixture SGC specimens with basalt fiber under different F-T cycles. The least square method of LM can be used to calculate the Prony series fitting parameters of the relaxation modulus of the asphalt mixture. The fitting parameters of the Prony series of the relaxation modulus of the asphalt mixtures incorporating SBS polymer and basalt fiber are summarized in Table 17, Table 18, Table 19, Table 20 and Table 21.

According to the fitting correlation coefficient in Table 17, Table 18, Table 19, Table 20 and Table 21, the fitting correlation coefficient *R*^2^ values of the Prony series of asphalt mixtures incorporating SBS polymer and basalt fiber at each test temperature is larger than 0.99 with a good fitting effect. At the same time, by comparing the Prony parameters of the generalized Maxwell model under F-T cycles at the same test temperature, it can be seen that the fitting parameters of the generalized Maxwell model of asphalt mixtures generally show a decreasing trend as the number of F-T cycles increases.

The fitting result means that the generalized Maxwell model can be accurately used to describe the viscoelastic behavior of the asphalt mixtures and provide a reference for the low-temperature performance of the asphalt mixtures. However, due to there being too many parameters of the generalized Maxwell model, the variation trend is not regular and intuitive, and the model parameters obtained by fitting are difficult to accurately reflect the changes in the viscoelastic properties of asphalt mixtures under F-T cycles.

2. Analysis of Relaxation Behavior of Asphalt Mixtures Incorporating SBS Polymer and Basalt Fiber Based on Burgers Model

In order to further analyze the effect of F-T cycles on the viscoelastic properties of asphalt mixtures incorporating SBS polymer and basalt fiber, the Burgers model was used to fit the relaxation modulus curves of asphalt mixture SGC specimens at different test temperatures under F-T cycles. The Levenberg–Marquardt (LM) method and general global optimization method were also used as the fitting methods. According to the fitting model, the four parameters (*G*_1_ and *G*_2_ are relaxation strengths, *ρ*_1_ and *ρ*_2_ are relaxation time) of the Burgers model of asphalt mixture specimens incorporating SBS polymer and basalt fiber at five temperatures (10 °C, 20 °C, 30 °C, 40 °C, and 50 °C) can be obtained and are summarized in Table 22, Table 23, Table 24, Table 25 and Table 26.

It can be seen that the overall level of the fitting correlation coefficient *R*^2^ of the Burgers model of asphalt mixtures at each test temperature is around 0.90, which is slightly smaller than that of the above generalized Maxwell model. Although the fitting effect of the Burgers model is slightly worse than the generalized Maxwell model, the Burgers model is still in good agreement with the creep compliance, which can be used to describe the relaxation modulus of the asphalt mixtures under various F-T cycles and test temperatures.

**Figure 9:**
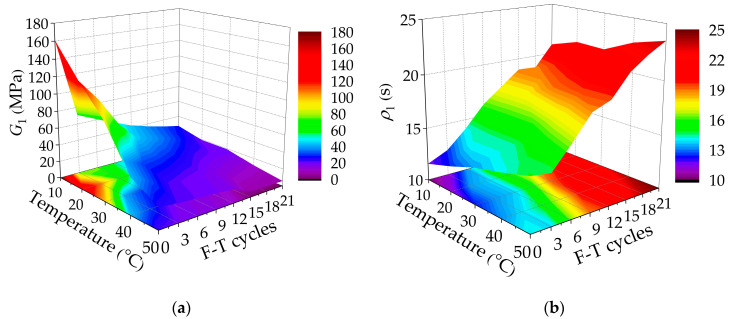

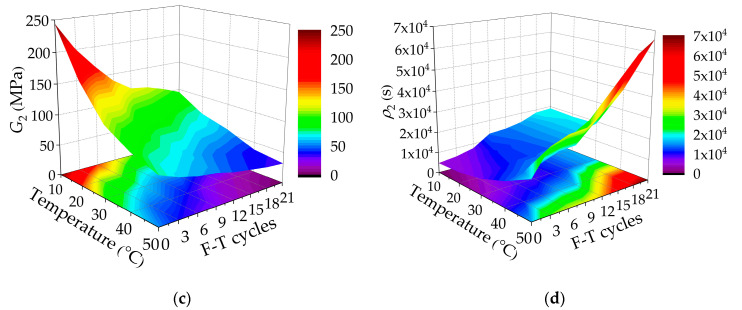
Burgers model parameters of relaxation moduli varying with temperatures and F-T cycles: (**a**) *G*_1_; (**b**) *ρ*_1_; (**c**) *G*_2_; and (**d**) *ρ*_2_.

Stress relaxation is an important method for evaluating the viscoelastic performance and crack resistance of asphalt mixtures. In order to better compare and evaluate the change of the relaxation performance of asphalt mixtures with test temperature and F-T cycles, the variation results of relaxation parameters with test temperature and F-T cycles are plotted based on the Burgers model, as shown in Figure 9.

Under the same F-T cycle, by comparing the parameters of the Burgers model at different test temperatures, it can be seen that the relaxation strengths *G*_1_ and *G*_2_ of asphalt mixtures decrease with the increase of test temperature, and its changing rate gradually slows down. Meanwhile, the relaxation time of the asphalt mixtures showed an upward trend with the increase of test temperature, and the changing rate gradually increased. At the same temperature, it can be seen that the relaxation strengths G1 and G2 of the Burgers model decrease with the increase of F-T cycles, and the relaxation times ρ1 and ρ2 increase as the F-T cycles increase. Moreover, the parameters of the Burgers model fluctuated to a certain extent during 9–12 F-T cycles. In addition, the temperature sensitivity of the relaxation properties of asphalt mixtures also decreases with F-T cycles.

As the test temperature increases, the asphalt mixtures gradually transfer from elasticity to viscoelasticity, and it gradually develops toward viscosity. Besides, under the F-T action, the internal structure of the asphalt mixtures changes significantly, which is mainly manifested by the increase in porosity and more connected pores. Then, there was damage inside under the influence of frost expansion force, and the relaxation modulus of the asphalt mixtures gradually decreased, which means that its ability to withstand stress is reduced and is conducive to crack resistance. Under the continuous action of F-T cycles, due to the weakening of the frost heave force, its internal damage decreases and the effect of water erosion is gradually obvious. The cohesion between the asphalt and aggregates weakens, and the changing rate of the relaxation strength slows down, while the changing rate of the relaxation time increases.

## 4. Conclusions

In this paper, the uniaxial compression creep test was used to analyze the static viscoelastic mechanical response of asphalt mixtures incorporating SBS polymer and basalt fiber under F-T cycles. The viscoelastic models were established and the viscoelastic parameters were analyzed to discuss the variation of viscoelastic characteristics. Based on this research, the following conclusions can be drawn:

- Test results showed that F-T cycles caused a larger creep deformation of asphalt mixture, and the creep compliance increased accordingly, which led to a decrease in the rut resistance of the asphalt mixture. Compared with lignin fiber, the incorporation of SBS polymer and basalt fiber can improve the resistance to high-temperature deformation of asphalt mixtures under F-T cycles.
- The instantaneous elastic modulus *E*_1_, instantaneous viscosity coefficient *η*_1_, delayed elastic modulus *E*_2_, and delayed viscosity coefficient *η*_2_ of the Burgers model showed a decreasing trend as the number of F-T cycles increases, and the resistance to deformation decreased significantly in the early stage of F-T cycles. At the same time, the improvement effect of the basalt fiber on asphalt mixtures in the early stage was better than that of the lignin fiber.
- Based on the conversion between relaxation modulus and creep compliance, the relaxation modulus of the asphalt mixtures gradually decreased with F-T cycles. By analyzing the slope and Burgers model parameters of the relaxation modulus, it can be seen that the relaxation time increased with F-T cycles, which will not be conducive to its internal stress dissipation. However, compared with lignin fiber, basalt fiber can improve its low-temperature crack resistance to a certain extent.

## Figures and Tables

**Table 1:** Technical parameters of styrene–butadiene–styrene (SBS)-modified asphalt.

Parameters	Unit	Values
Penetration	0.1 mm (@ 25 °C, 100 g, 5 s)	72
Ductility	cm (@ 15 °C, 5 cm/min)	45
Softening point	°C	60.5
Density	g/cm^3^	1.018
Flash point	°C	262
Rolling thin film oven test (RTFOT)		
Mass loss	%	−0.094
Penetration ratio	% (@ 25 °C)	66.9

**Table 2:** Technical properties of coarse fine aggregate.

Parameters		Unit	Values	Standard Limits
Crushing value		%	13.6	≤26
Los Angeles abrasion value		%	17.9	≤28
Apparent specific gravity	13.2 mm	—	2.836	≥2.6
	9.5 mm		2.805	
	4.75 mm		2.726	
Water absorption	13.2 mm	%	0.6	≤2.0
	9.5 mm		0.28	
	4.75 mm		0.7	

**Table 3:** Technical properties of fine aggregate.

Parameters	Unit	Values	Standard Limits
Apparent specific gravity	—	2.723	≥2.5
Water absorption	%	0.64	—
Angularity (flow time)	s	39.9	≥30
Sand equivalent	%	68	≥60

**Table 4:** Technical properties of mineral filler.

Parameters		Unit	Values	Standard Limits
Apparent density		t/m3	2.712	≥2.5
Hydrophilic coefficient		—	0.63	<1
Water content		%	0.3	≤1
Plastic index		%	2	<4
Granular composition	<0.6 mm	%	100	100
	<0.15 mm		92.5	90~100
	<0.075 mm		81.8	75~100

**Table 5:** Technical properties of basalt fiber.

Parameters	Unit	Values
Length	mm	6
Diameter	µm	13
Specific gravity	g/cm^3^	2.55~2.65
Tensile strength	MPa	≥3000
Elongation at break	%	3.2

**Table 6:** Prony series results of creep compliances under freeze–thaw (F-T) cycles (10 °C).

Parameters (MPa^−1^)	0 F-T	3 F-T	6 F-T	9 F-T	12 F-T	15 F-T	18 F-T	21 F-T
*J*_0_	1.50 × 10^−3^	2.69 × 10^−3^	2.73 × 10^−3^	4.05 × 10^−3^	5.38 × 10^−3^	3.98 × 10^−3^	5.50 × 10^−3^	6.20 × 10^−3^
*J*_1_	6.01 × 10^−4^	9.18 × 10^−4^	6.36 × 10^−4^	5.25 × 10^−4^	6.29 × 10^−4^	9.51 × 10^−4^	9.42 × 10^−4^	8.78 × 10^−4^
*J*_2_	6.69 × 10^−4^	2.48 × 10^−3^	1.32 × 10^−3^	2.00 × 10^−3^	1.87 × 10^−3^	1.58 × 10^−3^	1.36 × 10^−3^	1.27 × 10^−3^
*J*_3_	6.93 × 10^−4^	2.49 × 10^−3^	4.02 × 10^−3^	8.80 × 10^−4^	8.93 × 10^−4^	7.85 × 10^−4^	9.12 × 10^−4^	8.01 × 10^−4^
*J*_4_	2.40 × 10^−4^	5.54 × 10^−4^	6.06 × 10^−4^	6.08 × 10^−4^	6.67 × 10^−4^	1.03 × 10^−3^	9.89 × 10^−4^	7.73 × 10^−4^
*J*_5_	6.50 × 10^−4^	6.53 × 10^−4^	1.35 × 10^−3^	8.54 × 10^−4^	7.34 × 10^−4^	1.46 × 10^−3^	1.01 × 10^−3^	1.52 × 10^−3^
*J*_6_	7.53 × 10^−4^	6.02 × 10^−4^	9.08 × 10^−4^	6.36 × 10^−4^	5.39 × 10^−4^	1.33 × 10^−3^	8.42 × 10^−4^	1.02 × 10^−3^
*R* ^2^	0.99999	0.99999	0.99999	0.99999	0.99997	0.99997	0.99999	0.99999

**Table 7:** Prony series results of creep compliances under F-T cycles (20 °C).

Parameters (MPa^−1^)	0 F-T	3 F-T	6 F-T	9 F-T	12 F-T	15 F-T	18 F-T	21 F-T
*J*_0_	1.94 × 10^−3^	4.12 × 10^−3^	3.10 × 10^−3^	6.14 × 10^−3^	5.02 × 10^−3^	5.50 × 10^−3^	7.79 × 10^−3^	8.59 × 10^−3^
*J*_1_	9.99 × 10^−4^	1.39 × 10^−3^	2.48 × 10^−3^	1.90 × 10^−3^	2.03 × 10^−3^	2.18 × 10^−3^	1.93 × 10^−3^	1.75 × 10^−3^
*J*_2_	5.01 × 10^−3^	1.20 × 10^−3^	1.51 × 10^−3^	1.03 × 10^−3^	1.24 × 10^−3^	1.31 × 10^−3^	1.40 × 10^−3^	1.07 × 10^−3^
*J*_3_	7.47 × 10^−4^	2.33 × 10^−3^	1.49 × 10^−3^	1.22 × 10^−3^	1.25 × 10^−3^	1.08 × 10^−3^	1.29 × 10^−3^	1.11 × 10^−3^
*J*_4_	1.01 × 10^−3^	8.27 × 10^−4^	1.44 × 10^−3^	7.77 × 10^−4^	1.30 × 10^−3^	1.42 × 10^−3^	1.33 × 10^−3^	1.22 × 10^−3^
*J*_5_	1.51 × 10^−3^	6.66 × 10^−4^	1.17 × 10^−3^	7.46 × 10^−4^	1.48 × 10^−3^	2.02 × 10^−3^	1.44 × 10^−3^	2.11 × 10^−3^
*J*_6_	9.48 × 10^−4^	6.67 × 10^−4^	9.93 × 10^−4^	8.19 × 10^−4^	1.48 × 10^−3^	1.84 × 10^−3^	1.19 × 10^−3^	1.41 × 10^−3^
*R* ^2^	0.99999	0.99999	0.99999	0.99999	0.99999	0.99997	0.99999	0.99999

**Table 8:** Prony series results of creep compliances under F-T cycles (30 °C).

Parameters (MPa^−1^)	0 F-T	3 F-T	6 F-T	9 F-T	12 F-T	15 F-T	18 F-T	21 F-T
*J*_0_	3.34 × 10^−3^	5.23 × 10^−3^	6.66 × 10^−3^	8.66 × 10^−3^	6.91 × 10^−3^	9.22 × 10^−3^	7.50 × 10^−3^	1.04 × 10^−2^
*J*_1_	1.55 × 10^−3^	1.46 × 10^−3^	1.45 × 10^−3^	1.44 × 10^−3^	9.58 × 10^−4^	2.85 × 10^−3^	3.03 × 10^−3^	2.57 × 10^−3^
*J*_2_	1.60 × 10^−3^	1.16 × 10^−3^	9.99 × 10^−4^	1.07 × 10^−3^	1.51 × 10^−3^	1.55 × 10^−3^	1.85 × 10^−3^	1.87 × 10^−3^
*J*_3_	1.26 × 10^−3^	2.76 × 10^−3^	3.28 × 10^−3^	3.01 × 10^−3^	1.57 × 10^−3^	1.82 × 10^−3^	1.86 × 10^−3^	1.72 × 10^−3^
*J*_4_	1.62 × 10^−3^	1.05 × 10^−3^	8.64 × 10^−4^	1.01 × 10^−3^	1.34 × 10^−3^	1.17 × 10^−3^	1.94 × 10^−3^	1.59 × 10^−3^
*J*_5_	2.67 × 10^−3^	1.44 × 10^−3^	1.05 × 10^−3^	8.67 × 10^−4^	2.33 × 10^−3^	1.12 × 10^−3^	2.21 × 10^−3^	1.91 × 10^−3^
*J*_6_	1.07 × 10^−3^	1.93 × 10^−3^	1.40 × 10^−3^	1.18 × 10^−3^	1.90 × 10^−3^	1.23 × 10^−3^	2.20 × 10^−3^	1.78 × 10^−3^
*R* ^2^	0.99999	0.99998	0.99999	0.99997	0.99998	0.99999	0.99999	0.99999

**Table 9:** Prony series results of creep compliances under F-T cycles (40 °C).

Parameters (MPa^−1^)	0 F-T	3 F-T	6 F-T	9 F-T	12 F-T	15 F-T	18 F-T	21 F-T
*J*_0_	5.18 × 10^−3^	8.80 × 10^−3^	6.59 × 10^−3^	9.35 × 10^−3^	1.12 × 10^−2^	1.06 × 10^−2^	1.44 × 10^−2^	1.51 × 10^−2^
*J*_1_	1.34 × 10^−3^	1.46 × 10^−3^	2.61 × 10^−3^	2.31 × 10^−3^	2.29 × 10^−3^	9.68 × 10^−4^	9.88 × 10^−4^	1.18 × 10^−3^
*J*_2_	1.29 × 10^−3^	1.09 × 10^−3^	1.57 × 10^−3^	1.68 × 10^−3^	1.40 × 10^−3^	1.68 × 10^−3^	8.92 × 10^−4^	8.42 × 10^−4^
*J*_3_	1.53 × 10^−3^	3.06 × 10^−3^	1.30 × 10^−3^	1.55 × 10^−3^	1.45 × 10^−3^	1.18 × 10^−3^	1.44 × 10^−3^	7.84 × 10^−4^
*J*_4_	1.28 × 10^−3^	1.03 × 10^−3^	1.70 × 10^−3^	1.60 × 10^−3^	1.59 × 10^−3^	3.92 × 10^−3^	2.93 × 10^−3^	5.69 × 10^−3^
*J*_5_	2.09 × 10^−3^	1.82 × 10^−3^	2.2 × 10^−3^	1.72 × 10^−3^	1.84 × 10^−3^	2.45 × 10^−3^	1.93 × 10^−3^	2.75 × 10^−3^
*J*_6_	1.52 × 10^−3^	1.20 × 10^−3^	2.42 × 10^−3^	1.43 × 10^−3^	2.75 × 10^−3^	3.28 × 10^−3^	4.19 × 10^−3^	2.40 × 10^−3^
*R* ^2^	0.99999	0.99997	0.99997	0.99999	0.99999	0.99999	0.99984	0.99999

**Table 10:** Prony series results of creep compliances under F-T cycles (50 °C).

Parameters (MPa^−1^)	0 F-T	3 F-T	6 F-T	9 F-T	12 F-T	15 F-T	18 F-T	21 F-T
*J*_0_	7.82 × 10^−3^	7.69 × 10^−3^	1.04 × 10^−2^	9.89 × 10^−3^	1.18 × 10^−2^	1.65 × 10^−2^	2.05 × 10^−2^	2.32 × 10^−2^
*J*_1_	1.11 × 10^−3^	7.03 × 10^−4^	7.20 × 10^−4^	7.31 × 10^−4^	8.84 × 10^−4^	9.63 × 10^−4^	7.66 × 10^−4^	7.99 × 10^−4^
*J*_2_	1.01 × 10^−3^	1.22 × 10^−3^	1.05 × 10^−3^	1.53 × 10^−3^	1.95 × 10^−3^	1.34 × 10^−3^	6.12 × 10^−4^	5.61 × 10^−4^
*J*_3_	1.28 × 10^−3^	8.53 × 10^−4^	6.50 × 10^−4^	8.62 × 10^−4^	9.69 × 10^−4^	1.44 × 10^−3^	1.24 × 10^−3^	4.54 × 10^−4^
*J*_4_	9.74 × 10^−4^	2.85 × 10^−3^	3.05 × 10^−3^	2.48 × 10^−3^	2.55 × 10^−3^	2.31 × 10^−3^	1.68 × 10^−3^	1.45 × 10^−3^
*J*_5_	1.60 × 10^−3^	1.78 × 10^−3^	2.14 × 10^−3^	2.15 × 10^−3^	3.03 × 10^−3^	1.66 × 10^−3^	3.22 × 10^−3^	4.18 × 10^−3^
*J*_6_	1.92 × 10^−3^	2.38 × 10^−3^	1.41 × 10^−3^	3.51 × 10^−3^	3.53 × 10^−3^	4.69 × 10^−3^	2.37 × 10^−3^	2.01 × 10^−3^
*R* ^2^	0.99999	0.99994	0.99984	0.99999	0.99994	0.99983	0.99996	0.99929

**Table 11:** Fitting parameters of creep based on Burgers model under F-T cycles (10 °C).

Parameters	0 F-T	3 F-T	6 F-T	9 F-T	12 F-T	15 F-T	18 F-T	21 F-T
*E*_1_ (MPa)	119.66	83.75	71.69	60.67	51.18	50.98	45.38	38.99
*η*_1_ (MPa·s)	9.03 × 10^5^	8.82 × 10^5^	8.66 × 10^5^	7.70 × 10^5^	7.19 × 10^5^	5.21 × 10^5^	4.63 × 10^5^	4.62 × 10^5^
*E*_2_ (MPa)	246.66	210.17	199.39	185.29	177.80	146.78	136.42	134.55
*η*_2_ (MPa·s)	1.75 × 10^4^	1.61 × 10^4^	1.47 × 10^4^	1.25 × 10^4^	1.03 × 10^4^	8.23 × 10^3^	8.10 × 10^3^	5.95 × 10^3^
*τ* (s)	70.86	76.51	73.54	67.44	58.00	56.09	59.35	44.22
*R* ^2^	0.98704	0.98724	0.9747	0.98357	0.98447	0.95832	0.96978	0.96341

**Table 12:** Fitting parameters of creep based on Burgers model under F-T cycles (20 °C).

Parameters	0 F-T	3 F-T	6 F-T	9 F-T	12 F-T	15 F-T	18 F-T	21 F-T
*E*_1_ (MPa)	78.70	61.45	58.79	40.69	39.11	34.91	30.15	26.49
*η*_1_ (MPa·s)	6.58 × 10^5^	6.14 × 10^5^	5.75 × 10^5^	5.56 × 10^5^	5.52 × 10^5^	5.02 × 10^5^	4.91 × 10^5^	3.92 × 10^5^
*E*_2_ (MPa)	131.31	110.43	99.73	96.23	93.05	92.46	89.39	77.23
*η*_2_ (MPa·s)	1.01 × 10^4^	8.96 × 10^3^	7.58 × 10^3^	6.32 × 10^3^	5.50 × 10^3^	5.47 × 10^3^	5.35 × 10^3^	4.06 × 10^3^
*τ* (s)	77.00	81.17	76.04	65.64	59.12	59.15	59.90	52.55
*R* ^2^	0.98276	0.98828	0.98019	0.98289	0.96573	0.95832	0.96978	0.96341

**Table 13:** Fitting parameters of creep based on Burgers model under F-T cycles (30 °C).

Parameters	0 F-T	3 F-T	6 F-T	9 F-T	12 F-T	15 F-T	18 F-T	21 F-T
*E*_1_ (MPa)	44.58	30.91	27.12	23.67	23.29	21.19	20.46	17.66
*η*_1_ (MPa·s)	5.54 × 10^5^	5.27 × 10^5^	5.21 × 10^5^	5.12 × 10^5^	4.85 × 10^5^	4.77 × 10^5^	4.37 × 10^5^	3.80 × 10^5^
*E*_2_ (MPa)	97.05	84.64	79.47	64.96	59.53	56.02	52.38	48.37
*η*_2_ (MPa·s)	8.12 × 10^3^	7.15 × 10^3^	5.63 × 10^3^	4.50 × 10^3^	3.92 × 10^3^	3.48 × 10^3^	3.20 × 10^3^	2.80 × 10^3^
*τ* (s)	83.69	84.45	70.86	69.30	65.83	62.07	61.18	57.91
*R* ^2^	0.98019	0.97108	0.98357	0.98447	0.96642	0.98289	0.96573	0.96978

**Table 14:** Fitting parameters of creep based on Burgers model under F-T cycles (40 °C).

Parameters	0 F-T	3 F-T	6 F-T	9 F-T	12 F-T	15 F-T	18 F-T	21 F-T
*E*_1_ (MPa)	30.80	23.82	23.65	20.41	16.49	15.52	13.09	12.93
*η*_1_ (MPa·s)	4.35 × 10^5^	4.20 × 10^5^	4.12 × 10^5^	3.89 × 10^5^	3.82 × 10^5^	3.75 × 10^5^	3.66 × 10^5^	3.32 × 10^5^
*E*_2_ (MPa)	86.59	74.16	71.34	62.72	59.88	55.45	52.60	41.68
*η*_2_ (MPa·s)	7.44 × 10^3^	5.72 × 10^3^	4.70 × 10^3^	3.93 × 10^3^	3.15 × 10^3^	2.94 × 10^3^	2.43 × 10^3^	1.99 × 10^3^
*τ* (s)	85.91	77.08	65.92	62.58	52.60	53.02	46.13	47.79
*R* ^2^	0.96573	0.98447	0.95832	0.96978	0.96341	0.93558	0.93804	0.91875

**Table 15:** Fitting parameters of creep based on Burgers model under F-T cycles (50 °C).

Parameters	0 F-T	3 F-T	6 F-T	9 F-T	12 F-T	15 F-T	18 F-T	21 F-T
*E*_1_ (MPa)	22.73	20.56	17.27	16.94	14.80	11.25	10.09	9.36
*η*_1_ (MPa·s)	4.03 × 10^5^	3.64 × 10^5^	3.44 × 10^5^	3.43 × 10^5^	3.37 × 10^5^	3.05 × 10^5^	3.04 × 10^5^	2.89 × 10^5^
*E*_2_ (MPa)	75.58	72.81	63.04	62.10	57.52	54.08	49.60	40.88
*η*_2_ (MPa·s)	6.72 × 10^3^	5.50 × 10^3^	4.24 × 10^3^	3.50 × 10^3^	3.00 × 10^3^	2.52 × 10^3^	2.03 × 10^3^	1.78 × 10^3^
*τ* (s)	88.92	75.47	67.26	56.28	52.24	46.57	41.01	43.45
*R* ^2^	0.96341	0.93558	0.93804	0.94262	0.94858	0.9356	0.94006	0.92856

**Table 16:** Fitting parameters of relaxation moduli under F-T cycles.

Parameters		0 F-T	3 F-T	6 F-T	9 F-T	12 F-T	15 F-T	18 F-T	21 F-T
10 °C	Slope	2.633	2.483	2.372	2.308	2.222	2.183	2.146	2.075
	Intercept	−0.134	−0.103	−0.082	−0.073	−0.059	−0.061	−0.058	−0.047
	*R* ^2^	0.96341	0.97980	0.96223	0.97636	0.99236	0.99275	0.94615	0.96925
20 °C	Slope	2.465	2.338	2.318	2.154	2.106	2.044	1.995	1.933
	Intercept	−0.128	−0.111	−0.104	−0.069	−0.069	−0.061	−0.058	−0.047
	*R* ^2^	0.96341	0.98640	0.99184	0.96112	0.99306	0.96026	0.94633	0.96910
30 °C	Slope	2.285	2.125	2.092	2.015	1.999	1.978	1.932	1.870
	Intercept	−0.111	−0.073	−0.073	−0.069	−0.069	−0.066	−0.059	−0.058
	*R* ^2^	0.96341	0.96108	0.98009	0.99236	0.99281	0.97146	0.99305	0.95997
40 °C	Slope	2.093	2.009	1.965	1.916	1.818	1.730	1.667	1.614
	Intercept	−0.069	−0.061	−0.059	−0.058	−0.047	−0.034	−0.028	−0.020
	*R* ^2^	0.96341	0.96008	0.99277	0.94616	0.96920	0.96666	0.92164	0.92202
50 °C	Slope	1.974	1.869	1.805	1.782	1.717	1.638	1.580	1.529
	Intercept	−0.047	−0.035	−0.034	−0.034	−0.028	−0.021	−0.019	−0.012
	*R* ^2^	0.96341	0.96677	0.92200	0.92152	0.97847	0.97433	0.97992	0.91063

**Table 17:** Prony series results of relaxation moduli under F-T cycles (10 °C).

Parameters (MPa)	0 F-T	3 F-T	6 F-T	9 F-T	12 F-T	15 F-T	18 F-T	21 F-T
*E*_0_	133.86873	92.75010	116.00928	29.28880	103.41262	96.15385	90.17133	83.05648
*E*_1_	199.38382	131.56133	97.53875	40.11758	28.54813	17.13689	11.88436	8.73654
*E*_2_	141.61972	55.19087	61.25115	39.43923	13.13355	17.13688	11.88432	8.73651
*E*_3_	118.16312	42.77681	58.00132	22.37324	13.13355	17.13687	11.88415	8.73641
*E*_4_	8.93583	31.45977	27.54469	22.36709	0.00000	7.69466	6.88822	5.32675
*E*_5_	8.93583	26.39177	27.54418	19.43322	0.00000	7.69466	6.85077	4.98021
*E*_6_	0.00000	26.15596	9.19659	19.28879	0.00000	7.69466	6.84974	4.96011
*R* ^2^	0.99999	0.99999	0.99999	0.99999	0.99999	0.99999	0.99999	0.99999

**Table 18:** Prony series results of relaxation moduli under F-T cycles (20 °C).

Parameters (MPa)	0 F-T	3 F-T	6 F-T	9 F-T	12 F-T	15 F-T	18 F-T	21 F-T
*E*_0_	104.16667	96.24639	89.84726	82.98755	75.58579	69.68641	63.69427	59.95204
*E*_1_	42.53909	23.51554	95.99745	28.04605	20.88298	36.49830	7.24526	6.31619
*E*_2_	36.84454	23.51554	20.97286	12.86018	21.17662	8.38396	7.24526	6.31607
*E*_3_	36.84364	19.30993	20.97286	12.85980	20.88297	8.38396	6.28025	6.31568
*E*_4_	36.84300	19.30993	0.00000	0.01539	0.31390	0.26993	6.28025	3.79062
*E*_5_	28.58562	19.30992	0.00000	0.01529	0.31390	0.26991	6.28025	3.73603
*E*_6_	11.79649	10.77335	0.00000	0.01464	0.31387	0.26989	6.28025	3.50143
*R* ^2^	0.99999	0.99999	0.99999	0.99999	0.99999	0.99999	0.99999	0.99999

**Table 19:** Prony series results of relaxation moduli under F-T cycles (30 °C).

Parameters (MPa)	0 F-T	3 F-T	6 F-T	9 F-T	12 F-T	15 F-T	18 F-T	21 F-T
*E*_0_	83.40284	73.96450	68.58711	64.26735	59.70132	55.27916	50.65849	47.77831
*E*_1_	29.69837	13.24480	8.12975	5.90227	13.65544	6.23630	14.13191	6.28234
*E*_2_	29.69837	13.24468	8.12973	5.90226	10.12223	6.23629	9.75212	6.28217
*E*_3_	29.69837	13.24399	8.12971	5.90226	10.11532	6.23626	9.69079	6.28211
*E*_4_	12.97285	8.71569	7.99429	5.45144	9.97034	5.72323	9.19135	3.74926
*E*_5_	12.97285	8.69977	7.99426	5.45143	3.15877	5.72323	0.03322	3.70655
*E*_6_	12.97285	8.55138	7.99422	5.45141	0.00017	5.72322	0.00007	3.43863
*R* ^2^	0.99999	0.99999	0.99999	0.99999	0.99999	0.99999	0.99999	0.99999

**Table 20:** Prony series results of relaxation moduli under F-T cycles (40 °C).

Parameters (MPa)	0 F-T	3 F-T	6 F-T	9 F-T	12 F-T	15 F-T	18 F-T	21 F-T
*E*_0_	73.31378	63.25111	58.10575	53.10674	45.93477	42.30118	38.32886	35.53660
*E*_1_	13.82468	5.82089	10.37424	7.89825	2.81777	6.61848	7.46571	4.63338
*E*_2_	13.82468	5.82089	10.37421	7.89811	4.84421	6.61830	5.60238	4.63337
*E*_3_	13.82468	5.82089	10.37414	5.13976	4.84404	6.61822	3.50943	4.63337
*E*_4_	6.84339	5.36388	4.65701	4.02994	4.84400	4.89852	1.59079	0.97877
*E*_5_	6.84339	5.36388	4.65692	4.02993	2.81674	1.38077	0.00000	0.97120
*E*_6_	6.84339	5.36388	4.65691	4.02992	2.81646	0.53005	0.00000	0.96932
*R* ^2^	0.99999	0.99999	0.99999	0.99999	0.99999	0.99999	0.99999	0.99999

**Table 21:** Prony series results of relaxation moduli under F-T cycles (50 °C).

Parameters (MPa)	0 F-T	3 F-T	6 F-T	9 F-T	12 F-T	15 F-T	18 F-T	21 F-T
*E*_0_	65.83278	58.30904	52.57624	48.00768	41.00041	37.31343	33.25574	30.85467
*E*_1_	6.94926	9.11697	5.98578	7.23562	6.58507	2.51942	2.37462	1.98297
*E*_2_	6.94917	9.11692	5.98574	7.23551	6.58481	2.51924	2.37369	1.97919
*E*_3_	6.94915	9.11688	5.98194	7.23509	6.58353	2.51889	2.37213	1.97798
*E*_4_	4.52272	3.13252	2.40607	3.72059	2.29259	1.25890	1.04095	0.74760
*E*_5_	4.08528	3.13236	2.30322	3.16580	2.27938	1.25015	1.03873	0.71313
*E*_6_	3.52586	3.13234	2.28039	0.79910	2.24178	1.21082	0.98462	0.00000
*R* ^2^	0.99999	0.99999	0.99999	0.99999	0.99999	0.99999	0.99999	0.99999

**Table 22:** Fitting parameters of relaxation based on the Burgers model under F-T cycles (10 °C).

Parameters	0 F-T	3 F-T	6 F-T	9 F-T	12 F-T	15 F-T	18 F-T	21 F-T
*G*_1_ (MPa)	161.20	70.76	70.04	51.90	42.92	36.50	31.31	26.70
*ρ*_1_ (s)	11.56	12.42	14.12	16.15	17.54	18.93	18.85	20.92
*G*_2_ (MPa)	244.32	200.15	167.34	138.47	119.62	115.50	106.38	95.23
*ρ*_2_ (s)	4.71 × 10^3^	5.74 × 10^3^	7.76 × 10^3^	1.32 × 10^4^	1.37 × 10^4^	1.52 × 10^4^	1.85 × 10^4^	1.90 × 10^4^
*R* ^2^	0.98261	0.97915	0.97308	0.96399	0.96933	0.95756	0.95604	0.95226

**Table 23:** Fitting parameters of relaxation based on the Burgers model under F-T cycles (20 °C).

Parameters	0 F-T	3 F-T	6 F-T	9 F-T	12 F-T	15 F-T	18 F-T	21 F-T
*G*_1_ (MPa)	124.12	98.28	66.11	43.36	37.66	32.08	25.72	19.30
*ρ*_1_ (s)	12.40	14.13	14.50	16.03	17.52	18.93	19.84	21.72
*G*_2_ (MPa)	167.03	123.46	122.29	96.68	92.95	83.74	75.10	68.75
*ρ*_2_ (s)	5.35 × 10^3^	7.66 × 10^3^	1.08 × 10^4^	1.21 × 10^4^	1.37 × 10^4^	1.53 × 10^4^	1.73 × 10^4^	1.85 × 10^4^
*R* ^2^	0.96986	0.96864	0.96656	0.96295	0.95655	0.9574	0.95614	0.95225

**Table 24:** Fitting parameters of relaxation based on the Burgers model under F-T cycles (30 °C).

Parameters	0 F-T	3 F-T	6 F-T	9 F-T	12 F-T	15 F-T	18 F-T	21 F-T
*G*_1_ (MPa)	91.20	40.06	30.55	29.07	26.08	21.38	19.44	19.31
*ρ*_1_ (s)	13.37	14.09	14.48	16.04	18.95	19.75	20.64	21.59
*G*_2_ (MPa)	114.59	97.36	84.24	74.35	74.18	64.41	62.27	56.36
*ρ*_2_ (s)	7.67 × 10^3^	9.81 × 10^3^	1.19 × 10^4^	1.21 × 10^4^	1.32 × 10^4^	1.53 × 10^4^	1.73 × 10^4^	1.90 × 10^4^
*R* ^2^	0.96659	0.96784	0.96388	0.96922	0.96081	0.96285	0.95663	0.95614

**Table 25:** Fitting parameters of relaxation based on the Burgers model under F-T cycles (40 °C).

Parameters	0 F-T	3 F-T	6 F-T	9 F-T	12 F-T	15 F-T	18 F-T	21 F-T
*G*_1_ (MPa)	42.12	31.42	21.44	19.16	17.81	14.81	13.14	11.46
*ρ*_1_ (s)	14.04	14.56	15.44	17.39	19.47	20.49	20.91	22.80
*G*_2_ (MPa)	90.15	73.17	69.82	62.63	52.68	45.85	40.78	37.78
*ρ*_2_ (s)	1.21 × 10^4^	1.37 × 10^4^	1.53 × 10^4^	1.85 × 10^4^	1.90 × 10^4^	2.99 × 10^4^	3.91 × 10^4^	3.99 × 10^4^
*R* ^2^	0.95659	0.96923	0.95742	0.95606	0.9523	0.8993	0.89888	0.90389

**Table 26:** Fitting parameters of relaxation based on the Burgers model under F-T cycles (50 °C).

Parameters	0 F-T	3 F-T	6 F-T	9 F-T	12 F-T	15 F-T	18 F-T	21 F-T
*G*_1_ (MPa)	24.53	21.24	19.79	18.35	15.74	6.60	6.34	5.74
*ρ*_1_ (s)	14.86	14.58	16.57	18.74	19.41	21.41	22.59	23.52
*G*_2_ (MPa)	75.51	63.20	55.93	51.66	44.05	39.24	34.61	32.03
*ρ*_2_ (s)	1.84 × 10^4^	2.99 × 10^4^	3.29 × 10^4^	3.36 × 10^4^	3.91 × 10^4^	4.84 × 10^4^	6.09 × 10^4^	6.61 × 10^4^
*R* ^2^	0.95223	0.89921	0.89875	0.91024	0.91937	0.89402	0.9069	0.91699
